# Graph-facilitated resonant mode counting in stochastic interaction networks

**DOI:** 10.1098/rsif.2017.0447

**Published:** 2017-12-06

**Authors:** Michael F. Adamer, Thomas E. Woolley, Heather A. Harrington

**Affiliations:** 1 Wolfson Centre for Mathematical Biology, Mathematical Institute, University of Oxford, Oxford OX1 2JD, UK; 2 Cardiff School of Mathematics, Cardiff University, Senghennydd Road, Cardiff CF24 4AGs, UK

**Keywords:** chemical reaction networks, graph theoretic methods, sturm chains, resonant modes, quasi-cycles

## Abstract

Oscillations in dynamical systems are widely reported in multiple branches of applied mathematics. Critically, even a non-oscillatory deterministic system can produce cyclic trajectories when it is in a low copy number, stochastic regime. Common methods of finding parameter ranges for stochastically driven resonances, such as direct calculation, are cumbersome for any but the smallest networks. In this paper, we provide a systematic framework to efficiently determine the number of resonant modes and parameter ranges for stochastic oscillations relying on real root counting algorithms and graph theoretic methods. We argue that stochastic resonance is a network property by showing that resonant modes only depend on the squared Jacobian matrix *J*^2^, unlike deterministic oscillations which are determined by *J*. By using graph theoretic tools, analysis of stochastic behaviour for larger interaction networks is simplified and stochastic dynamical systems with multiple resonant modes can be identified easily.

## 1. Introduction

Systems of interacting agents are ubiquitous in the physical and biological sciences, from predator–prey models [1–5] to mathematical biology [6–8] and the vast field of chemical reaction networks [9–12]. Previous research highlights how resonant amplification of noise in stochastic interaction networks can lead to behaviour not anticipated from deterministic ordinary differential equation (ODE) models. In particular, cyclic behaviour, often termed ‘quasi-cycles’, may emerge in stochastic models where the deterministic counterpart does not show a Hopf bifurcation [1,13]. Stochastic effects have been responsible for unforseen dynamics, which are vital when agent copy numbers are low (e.g. ranging from the creation of thrombin that results in blood clots [14], to gene action [15], cell polarization [16], epidemics [17] and ecological systems [18–23]).

The main tools for investigating stochastic cycles are based on the direct calculation of power spectra for the constituents of the network from a Langevin equation [8,24,25], which demands knowledge of noise covariances. The determination of noise covariances requires extensive coarse graining, starting from a master equation formulation of the interaction system, and via weak noise expansions the deterministic equations, and a Fokker–Planck equation, can be calculated. Eventually, coarse graining allows the use of the simpler chemical Langevin equation [8]. In [13] an approximation procedure was presented which focussed on the eigenvalues of a matrix to predict quasi-cycles in a stochastic system. While the eigenvalue method is fast and elegant it can lead to false positives when investigating the number of oscillation frequencies named resonant modes. In this paper, we seek to streamline the coarse graining process by showing how the desired information, namely the number of resonant frequencies of a network, can be extracted from the deterministic equations only. We also find the parameter ranges associated with a number of resonant modes using graph theoretical approaches developed for chemical reaction networks. The techniques presented in this paper can be applied in a more general context than chemical reaction networks, namely to any stochastic dynamical system which can be modelled by a linear Langevin equation.

There is a large body of algebraic and graph theoretic techniques for studying deterministic mathematical models. Usually these mathematical models have a large number of parameters, typically one rate constant per interaction, and the model parameters are responsible for the dynamics of the system [10,26]. Past research focused successfully on exploiting the network structure of an interaction system for determining its dynamical behaviour, as network structure is a feature of a model and unaffected by the choice of rate constants [9–11]. In [10] it was shown how network structure can be used to determine whether a given chemical reaction network has stable steady states, a useful tool to rule out multistationarity in a network. More recently graph theoretical methods have been employed to show how network features such as feedback cycles can lead to oscillations and multistationarity in chemical reaction networks [9]. Recently, a generalized theory of Turing patterns has been developed exploiting network features [27]. Graph theoretical methods provide the additional advantage over the approach in [10], that they allow one to explore the bifurcation structure of the network. Despite the apparent advantage of using graph theoretical methods for the investigation of dynamical capabilities of interaction networks the graph-based investigation of stochastic models is still in its infancy.

In this paper, we provide an alternative route for calculating the resonant frequencies (and their parameter ranges) of stochastically driven oscillating systems. We use the existing techniques of Sturm's theorem and the graph theoretic methods of [9], but we combine them to be applicable to power spectral methods. Instead of solving the roots of a rational function of the power spectrum from the weak noise approximation, we investigate the maxima of this function. To do this, we adapt algebraic techniques (e.g. Sturm chains) and a graph theoretic formulation for finding the coefficients of the characteristic polynomial and thereby offering a methodology for studying stochastically driven oscillations without requiring excessive expansions. We will use the autocatalytic networks studied in [13,28,29] to illustrate the use of our method.

## 2. Methods

In this section, we introduce autocatalytic networks as the main example for our method and show how Sturm chains can be applied to find stochastic resonances. We conclude this section by finding sets of inequalities describing the phases of the three species autocatalytic network.

### 2.1. Autocatalytic networks and their power spectra

We illustrate our methodology by example of the autocatalytic systems discussed in [13], but the results presented in this paper can be applied to any dynamical system with a single stable steady state. Autocatalytic reactions form an important class of chemical reaction networks and many biological systems can be modelled by autocatalytic reactions. The defining feature of autocatalytic networks is that one reaction product is the catalyst for some other reactions and the system follows the general reaction scheme [13]
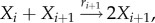

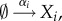

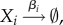
with
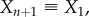
for a set of chemical species {*X*_1_, …, *X*_*n*_} and *i* = 1, …, *n*. In [13] a chemical master equation for the autocatalytic system was derived using the stochastic law of mass action [30]. Using a weak noise expansion [8] the deterministic equations for autocatalytic networks of *n* species were derived in [13]2.1
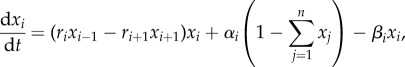
where *x*_*i*_ denotes the concentration of the *i*th species. Following the approaches of [13,28,29] we make the assumption that *r*_*i*_ = *r*_*j*_, *α*_*i*_ = *α*_*j*_ and *β*_*i*_ = *β*_*j*_ for all *i*, *j*. With these simplifications it can be shown that the system has a single steady state at2.2
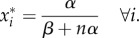
A linear stability analysis shows that the steady state is stable for all parameter values [13]. The deterministic equations represent the leading order of the expansion of the chemical master equation in the limit where the particle number *Ω* is large and at the next order we obtain a Fokker–Planck equation [8]. At steady state it is, however, simpler to use the equivalent representation of a chemical Langevin equation [1,8]2.3
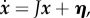
where bold quantities represent vectors, *J* is the Jacobian of equation (2.1) evaluated at the fixed point and ***η*** is a vector of Gaussian Markov processes. The covariances of the Markov processes 〈*η*_*i*_(*t*)*η*_*j*_(*t*′)〉 = *B*_*ij*_*δ*(*t* − *t*′) can be calculated from the Fokker–Planck equation. However, as will be shown in §2.2, it is not necessary for our methods to calculate noise covariances in detail. Only the fact that for white noise the covariances are constant will be used. Therefore, tedious expansions as traditionally used are not necessary, only knowing the deterministic equations suffices. Equation (2.3) determines the stochastic behaviour of autocatalytic networks at large, but finite *Ω*.

A useful tool to find oscillations in stochastic trajectories is the power spectrum 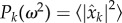 where 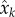 is the Fourier transform of the *k*th element of (2.3) and 〈 · 〉 denotes the average over a number of realizations [24]. The general form of the power spectrum of the *k*th species of any interaction network whose stochastic behaviour can be described by equation (2.3) is2.4
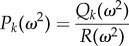
with2.5
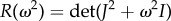
and2.6
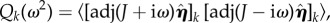
where *I* is the identity matrix, adj( · ) is the adjugate matrix, det( · ) is the determinant and 〈 · 〉 denotes the average. *R*(*ω*^2^) and *Q*_*k*_(*ω*^2^) are polynomials of degree *n* and *n* − 1, respectively, with *n* being the number of species in the network. Note that *R*(*ω*^2^) reduces to the characteristic polynomial of *J*^2^ if we let *ω*^2^ = − λ. Previous approaches proceeded by analysing all *n* rational functions (2.4) to determine the exact shape of the power spectra, and hence prove the existence of maxima. We will show how to determine the number of maxima and their parameter ranges by considering a single polynomial equation.

Stochastic oscillations manifest themselves as peaks in the power spectra which are closely linked to resonances. In analogy with the damped harmonic oscillator we define *ω*_*R*_ as a resonant frequency or resonant mode such that *R*(*ω*^2^_*R*_) is a minimum. Our definition implies that the resonant frequencies are properties of the underlying network structure, represented by *J*^2^, rather than the individual network constituents. Furthermore, our definition implies that every species in the network will have the same number of resonant modes, which is of course not true in general. In this paper, we assume that we only ever analyse networks with no disjoint subnetworks. Graph theoretically this condition translates into the graph of *J*, as defined in §3, being connected. If a network has disjoint subnetworks we can perform our analysis separately for each component of the graph of *J*. The polynomials *Q*_*k*_(*ω*^2^) can also suppress stochastic oscillations, but for the purpose of this paper, we use the approximation of [13] and assume the generic case where the number of modes is solely determined by *R*(*ω*^2^). Therefore, generically, the number of resonant modes is independent of the noise covariances 〈*η*_*i*_*η*_*j*_〉, even though resonance in interaction networks is a stochastic effect.

### 2.2. Sturm chains for counting the maxima of power spectra

We now turn to determine the number of resonant modes in a given network and show how parameter ranges for stochastic oscillations can be computed for the three species autocatalytic network. At resonance the polynomial *R*(*ω*^2^) has a minimum which translates into the condition2.7
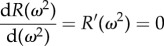
and, since the angular frequency *ω* is a real number, we are interested in finding all distinct, real, positive solutions to equation (2.7). A method to determine an upper bound of such solutions is given by ‘Descartes’ rule of signs' [31], which states that the maximum number of real, positive roots of a polynomial is given by the number of sign changes of consecutive non-zero coefficients, if the terms of the polynomial are ordered with descending variable exponent. Descartes' rule, however, only gives an upper bound and counts multiple roots as distinct roots.

An exact root counting algorithm is given through the computation of Sturm sequences and the use of Sturm's theorem [32]. For a univariate polynomial *p*(*x*) Sturm's theorem gives the number of distinct real roots in an interval (*a*, *b*] with *a* < *b*. To apply Sturm's theorem we compute a Sturm chain for *p*(*x*)2.8
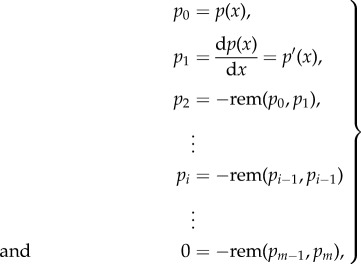
where rem( · , · ) is the remainder of the polynomial long division. Sturm's theorem proceeds by considering the signs of the Sturm chain *p*_0_, *p*_1_, …, *p*_*m*_ evaluated at the points *a* and *b*. Similarly to Descartes' rule the number of sign changes of *p*_0_(*a*), *p*_1_(*a*), …, *p*_*m*_(*a*) and *p*_0_(*b*), *p*_1_(*b*), …, *p*_*m*_(*b*) is counted which we denote as *σ*(*a*) and *σ*(*b*). The number of distinct real roots is simply *σ*(*a*) − *σ*(*b*). Letting *a* = 0 and *b* = ∞ gives the number of all positive, distinct, real roots. For small networks, especially the case *n* = 2, the number of real roots follows trivially from the quadratic formula and det(*A* + *xI*) = *x*^2^ + Tr(*A*)*x* + det(*A*), where Tr(*A*) is the trace. When turning to larger networks, however, Sturm chains become an invaluable tool.

### 2.3. Application to the three-species autocatalytic network

In this subsection, we will illustrate the usefulness of Sturm chains by example of a small system in the form of the *n* = 3 autocatalytic network. The three-species network is in fact the Rock–Paper–Scissors game for which stochastic simulations have been studied in [33]. In §4 we will show that the same reasoning can be extended easily to larger networks.

The *n* = 3 autocatalytic network is described by the deterministic equations2.9
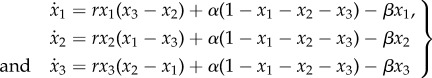
with a steady state2.10
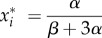
for every *i* = {1, 2, 3} and hence we will drop the index. The Jacobian of (2.9) evaluated at the steady state is2.11
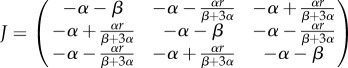
and, therefore, *R*(*ω*^2^) will be a degree three polynomial. Following the reasoning of the previous section we will need to compute Sturm chain for a quadratic polynomial, namely2.12
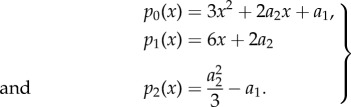
To find stochastic oscillations we will need to evaluate the Sturm chain at the points *x* = 0 and 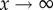,2.13a
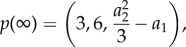
and2.13b
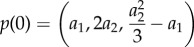
and their sign changes. For a stochastic resonance we will need the difference of sign changes to be either one or two. This follows from the fact that 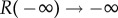 and 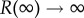 and, therefore, a maximum exists if and only if a minimum exists too and *ω*^2^ of the minimum will be larger than that of the maximum. As we are only interested in the minimum we need at least one sign change, hence for stochastic oscillations2.14
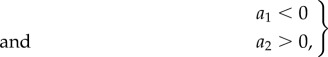
or2.15
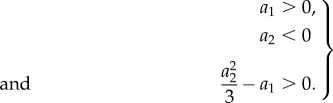
To relate the abstract notion of polynomial coefficients back to model parameters we will need to compute expressions for the coefficients *a*_*i*_.

From equations (2.14) and (2.15) it becomes apparent that often we only need to evaluate specific coefficients of *R*(*ω*^2^) rather than find the polynomial itself. Often, unless exact parameter ranges are needed, even fewer polynomial coefficients need to be considered due to some coefficients' inability to change sign, a feature easily identified from network motifs in the graph of *J*^2^. In the next section, we will outline a graph-based method to facilitate the finding of coefficients of *R*(*ω*^2^) based on [9].

## 3. Graph theoretic approach

**Figure 1.**
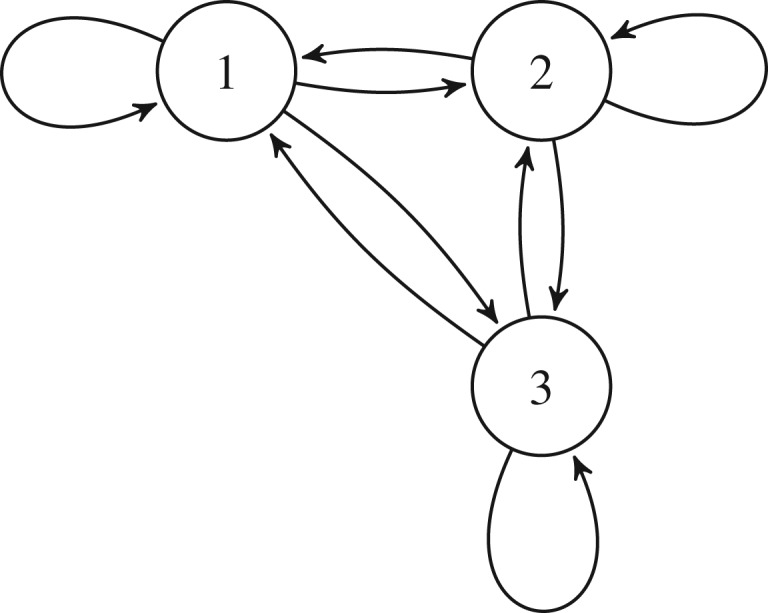
The directed graph associated with *J*^2^ of the *n* = 3 autocatalytic network. The edges have weights: 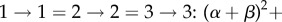 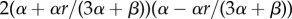, 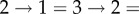 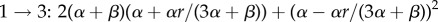 and 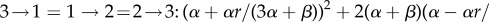 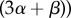.

In [9] a graph theoretic formula for the coefficients of the characteristic polynomial of the Jacobian of a chemical reaction network is given. A more general relation between the coefficients of a characteristic polynomial of a general square matrix *A* and the graph associated to *A* can be found in the earlier work of Maybee *et al.* [34]. We adapt the ideas of [9,34] for stochastic systems. We use the squared Jacobian *J*^2^, which is always a square matrix, as an adjacency matrix for a directed graph 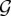. Define the vertex set 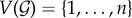 for an *n* species interaction network. Note that in contrast to the reasoning in [9] we no longer have a one to one correspondence between the vertex *i* and the chemical species *x*_*i*_ as we consider *J*^2^ rather than *J*. By definition there is an edge from vertex *i* to vertex *j* if *J*^2^_*ji*_ ≠ 0. The convention used in [9,34] is to only draw self loops if *A*_*ii*_ > 0, however, for convenience, we will always draw a self loop if *J*^2^_*ii*_ ≠ 0. It will become apparent that the choice to always draw self loops will only change the visual character of the graph but does not alter the calculations involved in any way. The reason for the convention in previous research was that often the diagonal elements *A*_*ii*_ had the same sign for any parameter values, e.g. the diagonal elements of (2.11) are always negative. As we will be dealing with the square of a matrix the diagonal elements will generically contain multiple terms and hence the sign will depend on the parameter values. Using these conventions we can define a vertex and an edge set which allow us to draw the directed graph for the *k* = 3 autocatalytic network as shown in figure 1.

In graph theory, a cycle *c* of length *k* in the graph 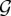 is defined as a series of distinct vertices {*v*_*i*_1__, …, *v*_*i*_*k*__} connected by edges *v*_*i*_1__*v*_*i*_2__, *v*_*i*_2__*v*_*i*_3__, …, *v*_*i*_*k*__*v*_*i*_1__ [35]. For a cycle *c* we denote *J*^2^[*c*] = (*J*^2^)_*v*_*i*_2___*v*_*i*_1____(*J*^2^)_*v*_*i*_3___*v*_*i*_2____ … (*J*^2^)_*v*_*i*_1___*v*_*i*_k____ which is the product of all the edge weights in the cycle. The cycles in an interaction graph such as figure 1 will be the fundamental building blocks for this graph theoretic approach. The graph in figure 1 has eight cycles, two of length three ({1, 2, 3} and {3, 2, 1}), three of length two ({1, 3}, {1, 2} and {2, 3}) and three length one cycles which are the self loops. In the method presented in this paper we are essentially dealing with complete directed graphs only, as, even though the Jacobian matrix of a chemical reaction system may be sparse, its square will generically be a dense matrix. Therefore, efficient cycle enumeration will be a non-trivial limitation of this method. However, computational experiments in SageMath [36] show that cycle enumeration is not a time limiting step in the calculation of phase diagrams.

Using the directed cycles of a graph 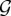 as building blocks we can define the concept of factors. A factor *f*_*k*_ of degree *k* of 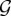 is a collection of pairwise disjoint cycles covering *k* distinct vertices. The number of cycles in a factor *f*_*k*_ is denoted by |*f*_*k*_|, which we shall call the cardinality of the factor. Hence, a graph can have multiple factors of the same degree, but with a vastly different number of cycles, e.g. the graph in figure 1 has a factor of degree three *f*^(1)^_3_ = {{1, 2, 3}} with |*f*^(1)^_3_| = 1 and a factor of the same degree *f*^(2)^_3_ = {{1}, {2}, {3}} with |*f*^(2)^_3_| = 3. Other factors of degree three can be built from the cycles.

Consider the characteristic polynomial 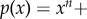 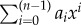 of a matrix *A*. We can now adapt a graph theoretic formula to find the coefficients *a*_*i*_, derived in [34] and applied to interaction networks in [9]. If the graph associated to the matrix *A* is 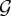 then3.1
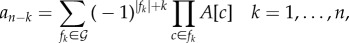
where in our example *A* = *J*^2^ and all other quantities are as previously defined.

While finding factors is trivial for small graphs the task can become computationally intractable for larger networks with more than six vertices. This is mainly due to the fact that no efficient algorithms for finding all possible factors of a graph exist. Additionally, the complexity is increased as the we are considering directed graphs which are generically complete. Finding all factors is the main bottleneck of the method.

Returning to equations (2.14) and (2.15) we need to find expressions for *a*_1_ and *a*_2_ and hence we will need to find all factors of degree two and one which are summarized in table 1. Therefore, by utilizing equation (3.1) we can find expressions for the coefficients *a*_1_ and *a*_2_,3.2
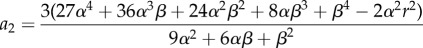
and3.3
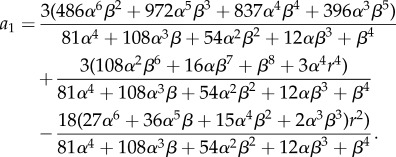
From the relations (2.14), (2.15) and (3.2), (3.3) we can plot a phase diagram of the system by either simplifying the resulting set of inequalities using cylindrical algebraic decomposition [37] or numerically by plugging in parameter values. A summary of the phases of the *n* = 3 autocatalytic network can be found in figure 4.

**Table 1.** A summary of all relevant factors in the *n* = 3 autocatalytic network graph.

*k* = 1	cycles	cardinality
*f*^(1)^_1_	{1}	1
*f*^(2)^_1_	{2}	1
*f*^(3)^_1_	{3}	1

*k* = 2	cycles	cardinality
*f*^(1)^_2_	{{1, 3}}	1
*f*^(2)^_2_	{{1, 2}}	1
*f*^(3)^_2_	{{2, 3}}	1
*f*^(4)^_2_	{{1}, {2}}	2
*f*^(5)^_2_	{{1}, {3}}	2
*f*^(5)^_2_	{{2}, {3}}	2

**Figure 2.**
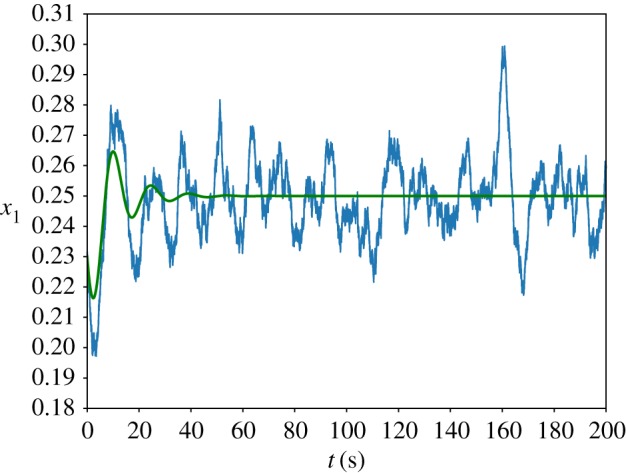
A trajectory of the *n* = 3 autocatalytic network with parameter values *α* = *β* = 0.1, *r* = 1 and *Ω* = 5000. The smooth, decaying curve is the numerical solution of the ODE system (2.9) and the oscillating trajectory is the stochastic trajectory. (Online version in colour.)

**Figure 3.**
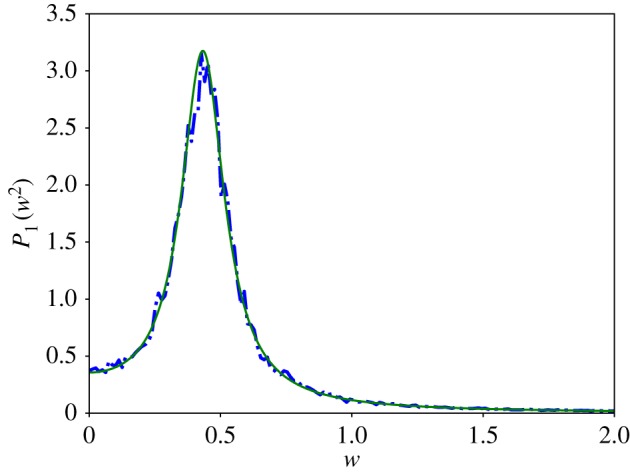
The power spectrum of the stochastic *n* = 3 autocatalytic network with parameter values *α* = *β* = 0.1, *r* = 1 and *Ω* = 5000. The smooth line represents the analytic curve, calculated from equation (14) in [13], and the dotted blue line is the average power spectrum of 200 simulations. Following [39], we normalized the spectra such that they have unit area. (Online version in colour.)

We simulated the trajectory of the stochastic *n* = 3 autocatalytic network in the parameter regime which satisfies condition (2.15) using Euler–Maruyama [38] integration of equation (2.3), figure 2 and plotted the power spectrum averaged over 200 repetitions. Our results can be found in figure 3 and show good agreement with the theoretical curve calculated in [13, equation (14)].

**Figure 4.**
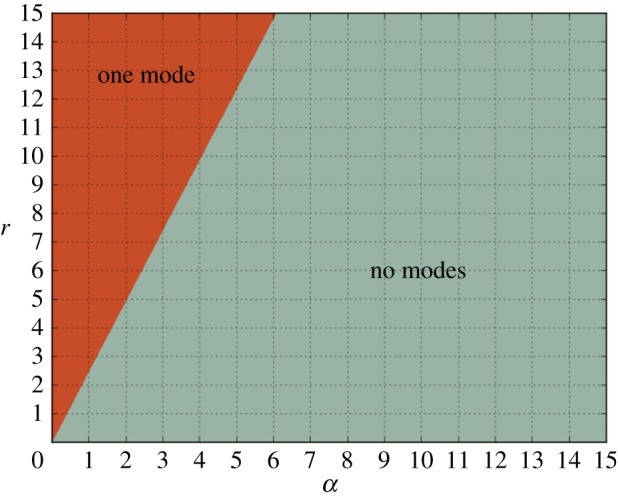
The phase diagram for the *α* = *β* slice of the parameter space of the *n* = 3 autocatalytic network. We identified two connected regions, one where stochastic oscillations are possible and one where the power spectrum is flat. (Online version in colour.)

## 4. Application to larger networks

In this section, we will show that our method can be applied with ease to larger networks by example of the *n* = 5 autocatalytic network. Traditional methods include the exact calculation of the power spectrum from the chemical Langevin equation [1] or approximations via the eigenvalues of the Jacobian matrix [13]. These tools have the capabilities of achieving the same results of finding the number of modes of a stochastic system; however, they are subject to serious drawbacks. Analytic expressions for the exact power spectra of a network can be calculated quickly in symbolic packages such as Mathematica [40] or SageMath [36]. However, such a calculation involves knowledge of the correlations of the Markov processes *η*_*i*_, 〈*η*_*i*_(*t*)*η*_*j*_(*t*′)〉 = *B*_*ij*_*δ*(*t* − *t*′), which are cumbersome to compute. Moreover, the full analytic form does not a priori give away any information about the number of stochastic modes of a system. To extract this information one would need to analyse the full rational function that is the power spectrum. While this is at best impractical, it can often be impossible and approximations need to be used. One such approximation was outlined in [13] and it focused on the pairs of complex conjugate eigenvalues of the Jacobian matrix. In [13] it was argued that the system will have a stochastic resonance if there exists a complex conjugate pair of eigenvalues λ_*i*_, λ*_i_ such that ℑ(λ_*i*_)^2^ − ℜ(λ_*i*_)^2^ > 0. While this is a quick and elegant method which also gives additional information about the relative intensities of resonances, it can lead to false positives. In particular, parameter regions for stochastic oscillations will be smaller than predicted as the approximation focuses at one factor of the characteristic polynomial of *J*^2^ at a time. In practice, however, other factors can ‘destroy’ the resonance. The method outlined in this paper will be able to exactly predict the parameter regions and the number of stochastic resonances; however, there are limitations on the network size which we will discuss in this section.

The five species autocatalytic network is described by the equations4.1
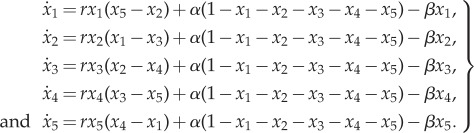
The steady state of the system is4.2
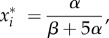
for every *i* = {1, …, 5}. The Jacobian evaluated at the steady state is4.3
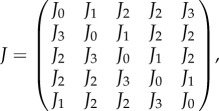
with4.4
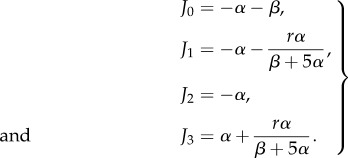
A linear stability analysis guarantees that system (4.1) is stable for any positive parameter values.

To determine the phase diagram we can follow the exact same procedure described above, namely

(1) compute the Sturm chain for a generic degree *n* − 1 polynomial;
(2) determine sets of inequalities on the Sturm coefficients to give resonances;
(3) compute the relevant coefficients of the polynomial using graph theoretic methods;
(4) use the information from steps 2 and 3 to plot a phase diagram.

In practice, however, this turns out to be cumbersome due to the vast number of sets of inequalities involved in step two, in addition to the quickly rising number of cycles and factors involved in step three. Furthermore, the explicit expressions for the Sturm coefficients can be cumbersome to work with. While it is possible to do step three on a computer, the inequalities involved in step two need to be formulated by hand. Therefore, to optimize the algorithm for automation we use the algorithm:

(1) compute the Sturm chain for a generic degree *n* − 1 polynomial;
(2) compute the relevant coefficients of the polynomial using graph theoretic methods;
(3) substitute parameter values and compute the number of real, positive roots;
(4) use this information to find the number of stochastic modes;
(5) plot the phase diagram.

**Figure 5.**
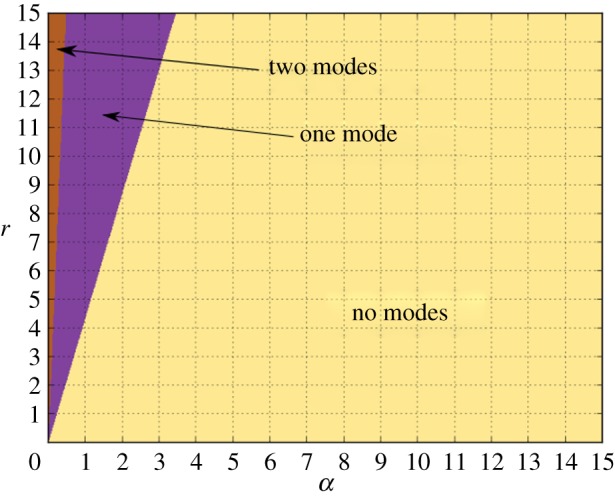
The phase diagram for the *α* = *β* slice of the parameter space of the *n* = 5 autocatalytic network. We identified three connected regions, one where there are two stochastic modes, one with only one mode and where no oscillations are possible. (Online version in colour.)

**Figure 6.**
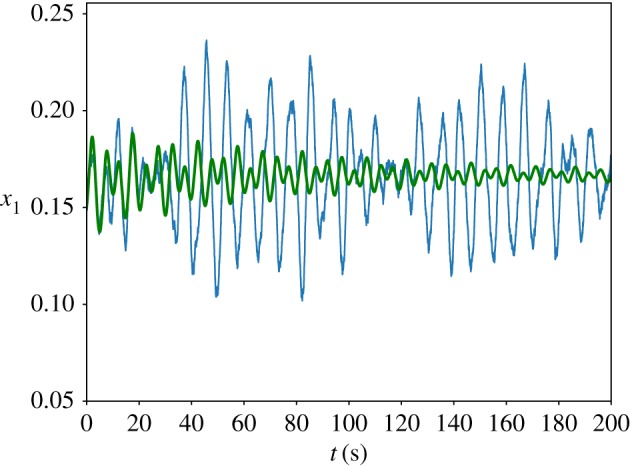
A trajectory of the *n* = 5 autocatalytic network with parameter values *α* = *β* = 0.01, *r* = 4 and *Ω* = 10 000. The smooth decaying curve is the numerical solution of the ODE system (4.1) and the blue curve is the stochastic trajectory. (Online version in colour.)

**Figure 7.**
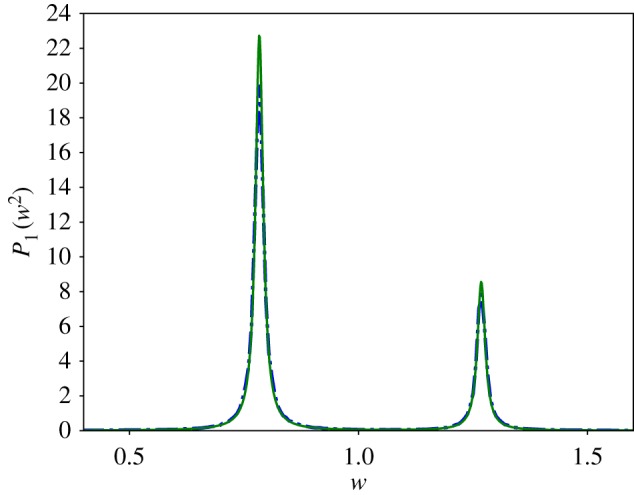
The power spectrum of the stochastic *n* = 5 autocatalytic network with parameter values *α* = *β* = 0.01, *r* = 4 and *Ω* = 10 000. The smooth green line represents the analytic curve, calculated from equation (14) in [13], and the dotted blue line is the average power spectrum of 500 simulations. Following [39], we normalized the spectra such that they have unit area. (Online version in colour.)

Step four is necessary due to the fact that a real, positive root could indicate a maximum or a minimum of *R*(*ω*^2^). This second algorithm can easily be implemented on a computer and phase diagrams can be calculated quickly. The only input required is a Jacobian evaluated at the steady state. We implemented our method in SageMath to plot a phase diagram for the *n* = 5 autocatalytic network in the *α* = *β* plane. Our results are summarized in figure 5. There are three regions in the phase diagram with two, one and no stochastic modes. We performed an Euler–Maruyama integration of equation (2.3) in the two-resonance regime and plotted the power spectrum. Our simulation results, summarized in figures 6 and 7, show that we accurately predict the number of resonant modes.

In principle, the method presented in this paper could be applied to networks of arbitrary number of species; however, there are a number of problems one encounters in networks with more species. The first problem is of fundamental nature and was already discussed in §3, namely the fact that we are generally dealing with complete, directed graphs and finding the factors of such a graph is a non-trivial task. The second issue is to do with numerical errors during computations. Substituting parameter values into the Sturm coefficients requires extensive floating point arithmetic and when the Sturm coefficients are small numerical errors will change the result. While our method should generally be robust, as only signs and sign changes are needed, as soon as one coefficient is small numerical fluctuations will become significant.

## 5. Conclusion

Numerous dynamical systems, which appear stable in a deterministic regime, can exhibit oscillatory behaviour when model stochasticity is accounted for. Such stochastically driven oscillations are likely to be missed in many applications. Here we have developed simple and general graph theoretic tools that allow ODE systems to be analysed as to the possibility of the occurrence of quasi-cycles. A vital tool to investigate stochastic oscillations is the power spectrum which is traditionally calculated from the Langevin equation. Current methods, however, require detailed knowledge of the underlying stochastic process which can be difficult to calculate. In this paper, we showed how resonance can be understood as a network property, independent of the noise correlations involved. We used Sturm chains to count the number of resonant modes and outlined a graph based method to determine parameter ranges in which stochastic oscillations occur. Future work will seek to extend the application of graph based methods to stochastic spatial systems such as stochastic Turing patterns in interaction networks.

## Supplementary Material

This is a Sage module which calculates phase diagrams using Sturm chains

## Data accessibility

The SageMath code for our method and examples is available as electronic supplementary material.
